# Factorial analysis of zinc serum levels, fatty acids, oxidative stress parameters and supplementation on assisted reproductive technology outcome

**DOI:** 10.5937/jomb0-53112

**Published:** 2025-07-04

**Authors:** Aleksandra Veselinović, Jelena Kotur-Stevuljevć, Aleksandar Stojsavljević, Dragana Bojović-Jović, Aleksandra Arsić, Marija Paunović, Vesna Vučić

**Affiliations:** 1 Research and Development Institute "Life Activities Advancement Institute", Cognitive Neuroscience Department, Belgrade; 2 Institute for Experimental Phonetics and Speech Pathology, Department of Speech, Language and Hearing Sciences, Belgrade; 3 University of Belgrade, Faculty of Pharmacy, Department of Medical Biochemistry, Belgrade; 4 University of Belgrade, Faculty of Chemistry, Innovative Centre, Belgrade; 5 Clinic for Gynecology and Obstetrics "Narodni Front", ART Department, Belgrade; 6 National Institute of Republic of Serbia, Centre of Research Excellence in Nutrition and Metabolism, Institute for Medical Research, Group for Nutritional Biochemistry and Dietology, Belgrade

**Keywords:** assisted reproduction, fatty acids, oxidative stress, zinc, potpomognuta reprodukcija, masne kiseline, oksidativni stres, cink

## Abstract

**Background:**

Infertility remains a prevalent global reproductive challenge, significantly affecting the lives of couples worldwide. The aetiology of infertility can be affected by various factors that exhibit possible relationships with one another. The study aimed to investigate factors that can interact with and influence the pregnancy outcome in couples undergoing assisted reproductive technology procedures.

**Methods:**

This retrospective study included 64 couples (64 men and their female partners) undergoing assisted reproductive technology procedures, having different pregnancy outcomes and lifestyle habits. Biomarkers of antioxidative and fatty acid status in the serum of both male and female partners, as well as the concentration of zinc in serum and seminal plasma of men, and their impact on assisted reproductive technology outcome were examined.

**Results:**

We grouped the parameters using principal component analysis and identified the three most contributing factors to the pregnancy achievement - seminogram parameters in males and redox status scores in female participants; supplementation with vitamin D, magnesium, and zinc; and serum levels of omega-3 and omega-6 fatty acids.

**Conclusions:**

The study concluded that seminogram parameters, intake of micronutrients, and levels of zinc, omega-3, and omega-6 fatty acids are contributing factors to the success of assisted reproductive technology. Further studies on a larger cohort are needed to confirm the predictive role of these factors on the success of assisted reproductive technology.

## Abstract (sr)

**Uvod:**

Neplodnost je i dalje preovlađujući globalni reproduktivni izazov koji značajno utiče na živote parova širom sveta. Na etiologiju neplodnosti mogu uticati različiti faktori koji pokazuju moguće odnose jedni sa drugima. Cilj istraživanja bio je da se ispitaju faktori koji mogu da međusobno deluju i utiču na ishod trudnoće kod parova koji su podvrgnuti postupku potpomognute reproduktivne tehnologije.

**Metode:**

Ova retrospektivna studija obuhvatila je 64 parova (64 muškaraca i njihovih partnerki) koji su bili podvrgnuti proceduri potpomognute reproduktivne tehnologije, sa različitim ishodom trudnoće i životnim navikama. Ispitivani su biomarkeri antioksidativnog i statusa masnih kiselina u serumu muških i ženskih partnera, kao i koncentracija cinka u serumu i u seminalnoj plazmi muškaraca; kao i njihov uticaj na ishod potpomognute oplodnje.

**Rezultati:**

Parametri su grupisani koristeći analizu glavnih komponenti i identifikovali tri faktora koji najviše doprinose uspešnosti oplodnje - parametri seminograma kod muškaraca i redoks status skor kod žena; suplementacija vitaminom D, magnezijumom i cinkom; i nivoi omega-3 i omega-6 masnih kiselina u serumu.

**Zaključak:**

Studija je zaključila da parametri seminograma, unos mikronutrijenata i nivoi Zn i omega-3 i omega-6 PUFA doprinose uspehu potpomognute reproduktivne tehnologije. Potrebne su dalje studije na većoj kohorti da bi se potvrdila prediktivna uloga ovih faktora na uspeh potpomognute reproduktivne tehnologije.

## Introduction

Infertility remains a global reproductive challenge, considerably affecting the lives of people worldwide. According to reports, 33–41% of cases of infertility are caused exclusively by a female factor, 25–39% by a male factor, and 9–39% by a combination of both male and female factor [1] [2]. Assisted reproductive technology (ART) includes fertility treatment in which either eggs or embryos are handled outside a female’s body to promote successful pregnancies and healthy offspring. Current ART procedures encompass in vitro fertilisation (IVF) with or without intracytoplasmic sperm injection (ICSI) [3].

Various exogenous and endogenous factors may contribute to a rising incidence of reproductive dysfunctions, including exposure to environmental stressors, different lifestyles, poor nutrition [4], and genetic and endocrine factors [5]. Nevertheless, in many cases, the causes of infertility are unclear. In line with this, the factors influencing ART outcomes are still not fully understood. Trace elements are critical in the male reproduction process [5]. One of the essential trace elements for male fertility is zinc (Zn), which has various roles in the male reproductive system, including regulating the process of spermatogenesis, epididymal sperm maturation, sperm structure and function, sperm motility, capacitation, fertilisation, and embryonic development [6]. Zinc also regulates oxidative stress and fatty acids (FA) metabolism [7].

Oxidative stress (OS) occurs when the concentration of prooxidants exceeds the antioxidant protection capacity [8] and contributes to many pathological conditions. The enzyme superoxide dismutase (SOD) plays a critical role in protecting cells from oxidative damage by catalysing the dismutation or disproportionation of the superoxide anion into hydrogen peroxide and oxygen [9]. Together with copper, Zn serves as a cofactor of SOD1, which is primarily found in cytoplasm, as well as for extracellular SOD, both of which protect cells and tissues from oxidative damage [10]. Consequently, Zn deficiency can impair the function of Cu/ZnSOD, leading to increased OS inthe body, including male reproductive tissues, which may negatively affect fertilisation success [11]. Furthermore, Zn deficiency and subsequent OS are linked to sperm DNA fragmentation, reduced sperm membrane integrity, apoptosis, antioxidant depletion, and ultimately, poor sperm quality and male infertility [12]. In women, biomarkers of oxidative stress, such as the lipid peroxidation index, reactive oxygen species levels and antioxidant enzyme activity, negatively affect the total antioxidant capacity. This highlights the potential importance and monitoring these biomarkers in the ART process [13].

Early reproductive events, such as oocyte maturation and embryo implantation, are influenced by FAs [14]. Previous studies of FA profiles in infertility aimed to provide insights into the mechanisms underlying fertility disorders and to explore potential therapeutic approaches, such as supplementation. However, the results remain inconsistent. Although adequate intake of omega-3 and/or omega-6 polyunsaturated fatty acids (PUFAs) has been emphasised to improve fertility rates, some studies have shown no significant difference in PUFA status between fertile and infertile women [15]. In men, FAs modulate oxidative stress, ROS production, and inflammatory processes in spermatogenesis [16]. Lower omega 3- PUFAs levels and higher omega 6 to omega-3 PUFA ratio have been observed in infertile men [17].

We aimed to examine possible cause-and-effect relationships among OS biomarkers, FA profiles, Zn concentration in both male and female serum and seminal plasma of men, and their potential predictive role in pregnancy outcomes in couples undergoing ART. Therefore, we aimed to determine whether certain endogenous and exogenous factors influence spermatogenesis and fertilisation in ART, aiming to improve ART success by addressing these factors.

## Materials and methods

### Study design and participants

The retrospective clinical study was conducted in the Department of Artificial Reproductive Techniques at the Clinic for Gynecology and Obstetrics »Narodni Front« in Belgrade, Serbia. All participants underwent face-to-face interviews conducted by trained medical doctors from the Clinic, covering demographic data, dietary habits, alcohol, tobacco and supplementation intake. Data on age, body mass index (BMI), occupation, medical history, place of residence, and lifestyle were also collected. The study received approval from the Ethics Committee of the Clinic for Gynecology and Obstetrics »Narodni Front« in Belgrade, Serbia (No. 05006-2022-19144, 4.11.2022.), and written informed consent was obtained from all participants.

This retrospective study enrolled 128 participants (64 couples – both males and females) undergoing ART procedures from January 2020 until December 2021. Half achieved successful pregnancy and delivery, while the other half did not conceive. The participants were divided into two groups: the P group (men and women who achieved pregnancy) and the N group (men and women who did not achieve pregnancy).

The inclusion criteria included healthy men aged 20–55 and women aged 20–45 who attended the Department of Artificial Reproductive Techniques at the Clinic for Gynecology and Obstetrics »Narodni Front« in Belgrade, Serbia.

The exclusion criteria for men included azoospermia, semen leukocyte count >1 x 10^6^/mL, failure to maintain at least 72 hours of abstinence before semen collection, genital infections (e.g. urethritis, prostatitis, sexually transmitted diseases), and systemic diseases (e.g. diabetes, cancer, autoimmune diseases). The exclusion criteria for women included a BMI over 30 kg/m^2^, chronic cardiovascular, metabolic, kidney, hepatic, malignant, and infectious diseases, premature follicular rupture, cryopreserved embryos not transferred in a fresh cycle, poor quality embryos, severe ovarian hyperstimulation syndrome, as well as missing or ectopic pregnancy outcome.

### Semen sampling and analysis

Semen samples were provided after self-masturbation into sterile containers at the Clinic unit after 72 hours of sexual abstinence. The samples were subsequently analysed in the same laboratory. After liquefaction at 37°C for 20 minutes, routine semen analysis – including liquefaction time, volume, pH, viscosity, sperm count, motility, and morphology – was performed, according to the WHO guidelines [18]. Clear, seminal plasma was separated from the sperm pellet by centrifugation at 3000 g for 30 minutes to ensure complete removal of the cellular components. The supernatants from another portion of the same sample were aliquoted and stored at -80°C until analysis.

The criteria for normal sperm parameters (normozoospermia) were as follows: sperm concentration ≥16×10^6^ million/mL of ejaculate; total sperm number >39 million per ejaculate; progressively motile sperms ≥30%, and sperm morphology ≥4%. Samples with sperm concentration ≥16×10^6^/mL of ejaculate, total sperm number >9 million per ejaculate, motility ≥30%, and morphology <4% were classified as teratozoospermic [18]. Samples with seminal parametersabnormalities other than teratozoospermia – such as asthenoteratozoospermic, oligoteratozoospermic and oligoasthenoteratozoospermic seminograms, were categorised as combined. Oligozoospermic and asthenozoospermic patients were not included in this study.

Thus, our male study group consisted of 64 men, divided into three groups: 1) 10 men with normozoospermia; 2) 34 men with teratozoospermia, and 3) 20 men with combined seminogram findings.

The procedures and guidelines of the ART Clinic for Gynecology and Obstetrics »Narodni Front« concerning infertility treatment have been previously outlined [19] [20].

To determine the levels of total FAs and antioxidative parameters, 5 mL of whole blood was drawn from all study participants, both men and their female partners. Blood samples were collected on the day of oocyte retrieval using Vacutainer tubes (BD Vacutainer Systems) and were allowed to coagulate for 30 minutes. The serum was then separated from the cells by centrifugation at 3000 × g, according to the manufacturer’s instructions. The samples were then stored at -20°C until analysis.

### Oxidative stress parameters measurements

All analyses of oxidative stress parameters were performed using the ELISA reader (Pharmacia LKB, Wien, Austria) or ILab 300+ (Instrumentation Laboratory, Milan, Italy).

### AOPP determination

Advanced oxidation protein products (AOPP) were measured using a reaction involving potassium iodide and glacial acetic acid, following the method outlined by Witko-Sarsat et al. [21]. Absorbance was measured at 340 nmm and the values between groups were compared. The concentration is expressed as mmol/L of serum.

### TOS determination

Total oxidative status (TOS) was assessed using the technique outlined by Erel [22]. This method involves oxidising the ferrous ion-o-dianisidine complex to ferric ion using oxidants within the sample. The resulting colour intensity was directly related to the total quantity of oxidant molecules. Absorbance was recorded at 560 nm. Concentrations of TOS in serum were expressed in units of μmol/L.

### PAB determination

The determination of PAB activity was conducted using a modified method developed by Alamdari et al. [23]. The modified PAB assay, utilising 3,30,5,50-tetramethylbenzidine (TMB) as a chromogen, was used to evaluate the prooxidant-antioxidant balance (PAB) [24]. This assay relies on the reaction between TMB, hydrogen peroxide, and antioxidants. Standard solutions were prepared by mixing varying amounts (0–100%) of 1 mmol/L hydrogen peroxide with 6 mmol/L uric acid. The absorbance was then measured at 450 nm. PAB values are expressed in arbitrary units U/L and indicate the quantity of hydrogen peroxide in the standard solution [25].

### IMA determination

Ischemia-modified albumin (IMA) levels were assessed in serum following the modified protocoldeveloped by Bar-Or et al. [26]. The assay procedure involved adding 0.1% cobalt chloride to serum, followed by an incubation period to facilitate binding between cobalt and protein molecules. Subsequently, dithiothreitol (DTT) was introduced, and after two minutes, the reaction was halted with a saline solution. The colour development resulting from the interaction with DTT was measured using a SPECTROstar Nano UV/VIS spectrometer, with values reported in absorption units (ABSU). Absorbance values higher than 0.400 indicate ischemia, while those less than 0.400 are negative for ischemia.

### SHG determination

Sulfhydryl (SH) group levels were quantified using Ellman’s method [27], which utilises the reaction between 2-nitrobenzoic acid and aliphatic thiols to produce a yellow-coloured p-nitrophenol. The quantification of SH groups was performed spectrophotometrically, assessing the absorbance of the resulting yellow product at 412 nm. The outcomes are reported in mmol/L of serum.

### TAS determination

The total antioxidative status (TAS) was assessed following Erel’s method [28]. This method involved the use of hydrogen peroxide in an acidic environment to oxidise reduced 2, 2-azino-bis (3-ethylbenzthiazoline-6-sulfonic acid) (ABTS), resulting in a change in the color of the reagent. The degree of colour change was directly related to the concentration of antioxidants present in the sample. Measurements were taken at 660 nm, and TAS concentration was expressed in mmol/L of serum.

### SOD determination

The assessment of superoxide dismutase (SOD) activity in serum followed the protocol established by Misra and Fridovich [29], with some adjustments. The method relies on the SOD capacity to hinder the spontaneous auto-oxidation of adrenaline in an alkaline condition. The enzyme’s activity was gauged by monitoring the absorption of the product formed through adrenaline oxidation at 480 nm. Results are expressed in U/L of serum.

### PON1 determination

The Richter and Furlong method [30] was used to evaluate the enzymatic activity of serum paraoxonase1 (PON1) by conducting kinetic measurements with paraoxon and diazinon-O-analog as substrates. The conversion rate of substrates was tracked kinetically at 405 nm, and the outcomes are reported in U/L of serum.

### The calculation of prooxy, antioxy and oxy score

The Z score statistics were used to estimate the cumulative impact of various ROSs on serum biomolecules. This method allows the simultaneous evaluation of parameters measured in different concentration ranges and units. Additionally, using Z score statistics enables concurrently assessment of the influence of different prooxidants and antioxidants. The Z score is calculated as the difference between the parameter value in the sample and the mean value of the same parameter in the general population, divided by the population standard deviation [31].

The oxy score represents two main aspects of oxidative stress: oxidative damage accumulation and reduced antioxidant defences. It is determined by subtracting the antioxy score (the mean Z value of antioxidant parameters: TAS and SHG) from the proxy score (the mean Z score of prooxidant parameters: TOS and PAB via the following formula:

[OXYSCORE = Mean (ANTIOXik – OXYim)n]

where n is the experimental group, i is the individual, k represents the parameters related to ANTIOX and m represents the parameters related to OXY biomarkers [32]. A higher oxy score signifies weaker antioxidative protection and a dominance of prooxidative processes.

### Fatty acids measurements

Fatty acids from total plasma lipids were isolated by a direct transesterification method with 3N HCl in methanol at 85°C for 60 minutes. The fatty acid methyl esters were extracted with hexane and evaporated in nitrogen to dryness [33]. The samples were analysed by gas chromatography using a Shimadzu gas chromatograph GC 2014 (Kyoto, Japan) equipped with Rtx 2330 column (60 m × 0.25 mm i.d., film thickness 0.25 mm, Restek, USA). The gas chromatography conditions included a helium flow rate of 1 mL/min, an initial temperature of 140°C held for 10 min, and a programmed increase at 3°C/min to a final temperature of 220°C, which is kept for 20 min. Individual FAs were identified using standard mixtures PUFA-2 and Supelco 37 Component FAME Mix (Sigma-Aldrich, Germany), and the results were expressed as percentages of total identified FAs. FA desaturase activities (SCD-18, SCD-16, D6D, D5D) and elongase activity were estimated by calculating the ratios of relative abundances of specific FA pairs [34].

### Quantification of Zn

A singular standard solution of Zn at a concentration of 10 mg/L was used to generate six intermediate standard solutions. The resulting calibration curve exhibited a linearity exceeding 0.999. A Ge internal standard solution at a concentration of 10 mg/L was utilised following a final dilution to 10 μg/L to address matrix interferences. This solution was uniformly dispensed across blanks, standard solutions, and samples via a secondary channel of the peristaltic pump. Zn quantification was conducted using inductively coupled plasma mass spectrometry (ICP-MS, iCAP Qc, Thermo Scientific, UK) in the helium (He) mode. The accuracy of the analytical technique was verified using the standard reference materials (SRM) (SERO210105, Level-1, supplied by Seronorm, Sero AS, Norway); the quantified values of ^66^Zn agreed from 94.1 to 102.7% with the declared values of the SRM, indicating the high accuracy of the ICP-MS technique used.

### Statistical analysis

All samples were checked from normality distribution data. Subsequently, comparisons were made between couples who achieved pregnancy (P group) and those who did not (N group). The Kolmogorov-Smirnov test was used to assess normality distribution. Data are shown as mean ± SD for continuous variables with normal distribution, while categorical variables are presented as relative and absolute frequencies. Since the distributions for certain parameters in the tables were skewed, data for those parameters were presented as median and interquartile range (IQR). Statistical analysis involved comparing normally distributed continuous variables using the Student’s t-test and non-normally distributed continuous variables using the Mann-Whitney U test. The analysis revealed no significant differences between men from the P group and men from the N group and between women from the P group and women from the N group.

The next step in data analysis involved principal component analysis (PCA) with varimax-normalised rotation to reduce the number of examined variables to fewer factors. The processed data included normally distributed variables and skewed distribution after log transformation. An extracted factor was determined based on eigenvalues >1. Variables with factor loadings ≥0.5 were used for factor interpretation. Scores were calculated for factors with eigenvalues >1, and those factors were included as independent variables in further analysis.

The analyses were performed with the SPSS statistical package (v.22.0; IBM, Chicago, Illinois, USA). The differences P < .05 were considered significant.

We used Z-score statistics to estimate the cumulative impact of various ROSs on serum biomolecules. This method simultaneously evaluates parameters measured in different concentration ranges and units. Additionally, using Z-score statistics enables the simultaneous assessment of the influence of different prooxidants and antioxidants. The Z score is calculated as the difference between the value of the parameter in the sample and the mean value of the same parameter in the general population, divided by the population SD. The oxy score is determined by subtracting the antioxidant score (the mean Z value of antioxidant parameters: TAS, SHG, and PON1) from the prooxidant score (the mean Z score of prooxidant parameters: AOPP, TOS, and PAB). A higher oxy score signifies weaker antioxidative protection and a dominance of prooxidative processes.

## Results

Table 1 presents the demographic characteristics, BMI, infertility duration, seminogram types, andseminogram related parameters in men from the P and N groups. The mean age of men in the P group was 38.9 ± 4.08 years with a median BMI was 26.5 kg/m^2^. In the N group, the mean age of men was 39.3 ± 6.01 years, and their median BMI was 26.6 kg/m^2^ (Table 1).

**Table 1:** Demographic characteristics of men from the P and N groups. Abbreviations: P group, men (and women) who achieved pregnancy; N group, men (and women) who did not achieve pregnancy; BMI, body mass index (kg/m2); Ns, non-significant.

Men	P group, n=31	N group, n=33	*p*-value
Age, years	38.9±4.08	39.3±6.01	Ns
BMI, kg/m^2^	26.5 (2.48)	26.6 (2.91)	Ns
Infertility, years	7 (5)	6 (9)	Ns
Sperm pH	7.80 (0.30)	7.80 (0.30)	Ns
Sperm concentration (x 10^6^)	37.2±24.4	34.6±23.8	Ns
Total sperm number	75.8 (97.8)	65.7 (118.5)	Ns
Sperm progressive motility (%)	35 (6)	35 (6.50)	Ns
Sperm morphology/normal forms (%)	18 (17)	18 (16.5)	Ns
Normozoospermia (%)	16.1	15.2	Ns
Teratozoospermia (%)	48.4	57.6	Ns
Combined seminogram (%)	35.5	27.3	Ns

Table 2 displays the demographic characteristics, BMI, and hormonal-related parameters in women from the P and N groups. The mean age of women in the P group was 36.3 ± 4 years and their median BMI was 22.6 kg/m^2^; while the mean age of women from the N group was 38.2 ± 3.9 years, and their median BMI was 22.0 kg/m^2^ (Table 2). These and other parameters were similar in both groups, showing that groups were well balanced.

**Table 2:** Demographic characteristics of women from the P and N groups. Abbreviations: P group, women who achieved pregnancy; N group, women who did not achieve pregnancy; BMI, body mass index (kg/m^2^); FSH, Folicle-Stimulating Hormone, LH, luteinising hormone; AMH, Anti-Müllerian hormone; E2, Estradiol; P4, Progesterone; Ns, non-significant. Normally distributed continuous variables are presented as mean ± SD; variables with a skewed distribution are presented as median and (IQR). Continuous variables are compared using the Student’s t-test or the Mann-Whitney U test, while categorical variables are compared using the Chi-square test.<br>**p < 0.01, ***p < 0.001.

Women	P group, n=31	N group, n=33	p-value
Age, years	36.3±4	38.2±3.9	Ns
BMI, kg/m^2^	22.6 (4.32)	22.0 (4.09)	Ns
Infertility, years	7 (5)	6 (9)	Ns
FSH, mIU/mL	6.42 (2.86)	7.30 (4.12)	Ns
LH, mIU/mL	5.50 (3.20)	4.90 (2.88)	Ns
AMH, ng/mL	1.80 (2.44)	1.22 (1.63)	Ns
E2, pg/mL	111 (54.0)	130 (58.5)	Ns
P4, ng/mL	1.17 (1.15)	1.60 (0.62)	Ns
Mature oocyte number	7.71±3.92	4.79±3.22	<0.01**
Number of fertilized oocyte	6.52±3.41	3.52±2.18	<0.001***

Normally distributed continuous variables are presented as mean ± SD; variables with a skewed distribution are presented as median and interquartile range (IQR). Continuous variables are compared using the Student’s t-test or the Mann-Whitney U test.

No significant differences were found in the hormonal levels of women from the P and N groups. However, there is a significant difference in the number of mature oocytes and in the number of fertilised oocytes in women from the P and N groups based on the pregnancy achievement criteria (Table 2).

There were no statistically significant differences in tobacco smoking between men and women from the P and N groups (Table 3). Data collected on supplements use – including Zn, vitamin D, magnesium (Mg), and omega-3 PUFA – showed no significant differences in men from the P and N groups. However, an important difference was observed in the use of antioxidants such as vitamin C or alpha-lipoic acid, with 4 men (13.3 %) in the P group using them, compared to none in the N group (0%), as indicated by the Chi-square test results (*χ^2^* = 4.69, *df*= 1, *r* = .07, *p*= .03*) (Table 3). No significant differences were found in vitamin and antioxidant use between women from the P and N groups (Table 3).

**Table 3:** Prevalence of smoking and supplement use in men and women from the P and N groups. Abbreviations: P group, men and women who achieved pregnancy; N group, men and women who did not achieve pregnancy; PUFA, polyunsaturated fatty acids.<br>Categorical variables were compared by Chi-square test. Variables are presented as median and (IQR).<br>*p < 0.05 vs P group (men).

Parameter	Men		Women	
	P group, n=31	N group, n=33	P group, n=31	N group, n=33
Tobacco smoking, %	8 (25.8 %)	13 (39.4 %)	6 (19.4 %)	9 (28.1 %)
Zinc use %	21 (67.7 %)	20 (60.6 %)	21 (70.0 %)	22 (68.8 %)
Vitamin D use %	18 (58.1 %)	17 (51.5 %)	25 (83.3 %)	24 (75.0 %)
Magnesium use %	17 (54.8 %)	16 (48.5 %)	22 (73.3 %)	21 (65.6 %)
Omega-3 PUFA use %	4 (12.9 %)	7 (21.2 %)	15 (50.0 %)	14 (43.8 %)
Antioxidant use %	4 (13.3 %)	0 (0%)*	8 (26.7 %)	4 (12.5 %)

Table 4 shows that there were no significant differences in the circulating levels of OS biomarkers in the serum of men and women from the P and N groups. The omega-3 and omega-6 PUFA levels were similar in both male and female participants, regardless of the use of omega-3 supplements. Additionally, no differences were found in Zn concentrations in seminal plasma between men from the P and N groups. Interestingly, men in the P group had significantly higher serum Zn levels (787 μg/L) than those in the N group (672 μg/L) (Table 4).

**Table 4:** Circulating levels of oxidative stress parameters in participants from the P and N groups. Abbreviations: P group, men and women who achieved pregnancy; N group, men and women who did not achieve pregnancy; AOPP, advanced oxidation protein products; PAB, prooxidant-antioxidant balance; TOS, total oxidant status; SHG, sulphydryl groups; TAS, total antioxidant status; SOD, superoxide dismutase; PON1, paraoxonase 1; IMA, ischemia-modified albumin; TOSz total oxidant status score, PABz, prooxidant-antioxidant balance score; SHGz, sulphydryl groups score, TASz, total antioxidant status score; PUFA, polyunsaturated fatty acids. Normally distributed continuous variables are presented as mean ± SD; variables with a skewed distribution are presented as median and (IQR). Comparisons between groups were conducted using the Student’s t-test or the Mann-Whitney U test.<br>**p < 0.01.

Variable	Men		Women	
	P group<br>n=31	N group<br>n=33	P group<br>n=31	N group<br>n=33
AOPP, μmol/L	79.0 (46.8)	93.2.5 (49.4)	77.0 (34.7)	64.1 (48.3)
TOS, μmol/L	34.1 (77.2)	50.5 (100.6)	81.4 (87.1)	45.6 (69.6)
PAB, U/L	50.2 (13.32)	50.8 (18.3)	68.7 (40.0)	75.4 (26.4)
SHG, mmol/L	0.421 (0.159)	0.432 (0.274)	0.360 (0.169)	0.355 (0.122)
TAS, μmol/L	509±182	499±183	601±166	591±177
SOD, U/L	94.0 (19.3)	94.0 (22.0)	84.0 (19.0)	85.0 (23.8)
PON1, U/L	345 (581)	195 (317)	231 (404)	335 (481)
IMA, ABSU	0.658 (0.234)	0.745 (0.339)	0.631 (0.153)	0.632 (0.254)
n-3 PUFA (mol%)	2.64 (1.71)	2.86 (1.87)	2.62 (0.82)	3.08 (1.14)
n-6 PUFA (mol%)	38.2±2.91	37.3±2.90	36.7±4.50	36.8±2.68
Seminal plasma Zn concentration (μg/L)	77388±8106	65057±9449	/	/
Serum Zn concentration (μg/L)	787 (729)	672 (150)**	/	/

**Figure 1:**
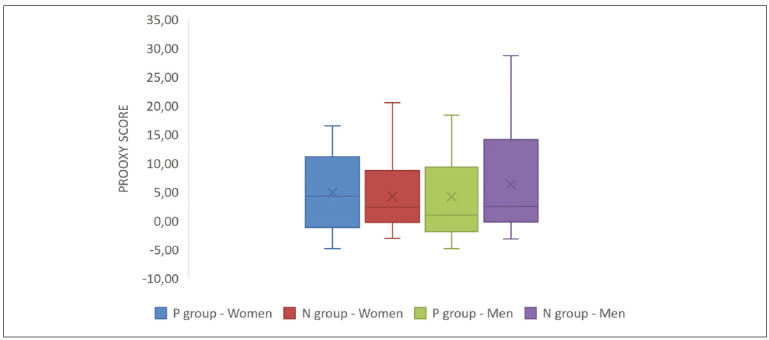
Prooxy score in men and women from the P or N group. P – achieved pregnancy, N – non-achieved pregnancy. A. Prooxy, B. antioxy and C. oxy scores scores in men and women from the P or N group. P – achieved pregnancy, N – non-achieved pregnancy. The boxplot displays the median and interquartile range, covered by the outliers

**Figure 2:**
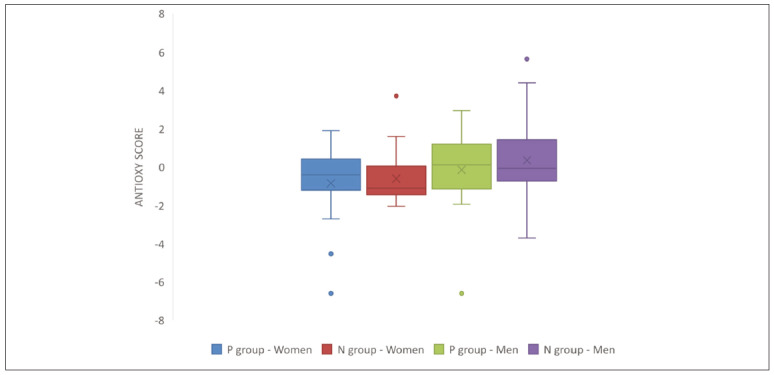
Antioxy score in men and women from the P or N group. P – achieved pregnancy, N – non-achieved pregnancy.

**Figure 3:**
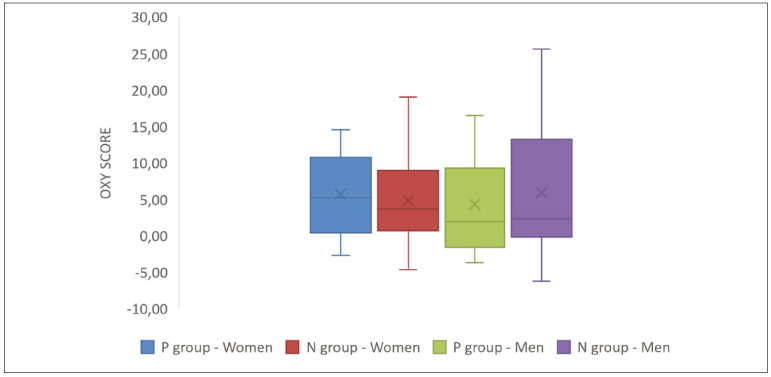
Oxy score in men and women from the P or N group. P – achieved pregnancy, N – non-achieved pregnancy.

The prooxy, antioxy and oxy scores are presented in Figure 1. Figure 2 Figure 3

We used the Principal Component Analysis (PCA) methodology to reduce the number of original variables and detect the relationships between them. As the PCA methodology combines initial predictors in smaller numbers of sets and allows for a better understanding of how each variable is associated and related to another variable, our results identified a combination of factors involved in pregnancy achievement outcomes in both men and women.

Before conducting PCA, the suitability of the data for factor analysis was assessed. Examining the correlation matrix revealed many coefficients of values 0.3 and above. The value of the Kaiser-Meyer-Olkin (KMO) indicator was 0.523 for men and 0.528 for women. Also, Bartlett’s sphericity test reached statistical significance *p*<0.001, indicating the correlation matrix’s factorability.

PCA revealed the presence of three components with characteristic eigenvalues greater than 1, explaining 19.5%, 15.6% and 12.9% and of the variance (cumulative 48%) in men’s data (Table 5). These components were seminogram parameters (seminogram type, sperm morphology, sperm concentration, sperm progressive motility); vitamin/mineral/fatty acids intake (Mg intake, Zn intake, vitamin D intake and omega-3 PUFA serum concentration); and fatty acids, sperm related and Zn related factor (omega-6 PUFA serum concentration, seminal plasma Zn concentration, sperm pH, serum Zn concentration). For women’s data (Table 6), the variance explained was 26.7%, 24.6% and 19.9% (cumulative 71.2%) for the components of redox status related score (oxy score, prooxidant score); vitamin/antioxidant use (vitamin D intake, Zn intake, Mg intake); PUFA and antioxidant score related factor (omega-6 PUFA serum concentration, omega-3 PUFA serum concentration, antioxidant score).

**Table 5:** Extracted men-related factors by PCA. Abbreviations: PCA, Principal Component Analysis; PUFA, polyunsaturated fatty acids

Factors	Included Variables With Loadings	Factor Variability (%)
Seminogram parameters	Seminogram type (-0.870)<br>Sperm morphology (0.783)<br>Sperm concentration (0.752)<br>Sperm progressive motility (0.690)	19.5
Vitamin/mineral/fatty acids intake	Magnesium intake (0.805)<br>Zinc intake (0.802)<br>Vitamin D intake (0.680)<br>Omega-3 PUFA serum<br>concentration (0.546)	15.6
Fatty acids, sperm-related and Zn<br>related factor	Omega-6 PUFA serum<br>concentration (0.717)<br>Seminal plasma Zn<br>concentration (0.618)<br>Sperm pH (-0.506)<br>Serum Zn concentration (0.505)	12.9
**Sum**		**48**

**Table 6:** Extracted women-related factors by PCA. Abbreviations: PCA, Principal Component Analysis; PUFA, polyunsaturated fatty acids

Factors	Included Variables With Loadings	Factor Variability (%)
Redox status-related score	Oxy score (0.990)<br>Prooxidant score (0.990)	26.7
Prooxidant score (0.990)	Vitamin D intake (0.780)<br>Zinc intake (0.773)<br>Magnesium intake (0.756)	24.6
PUFA and antioxidant score related factor	Omega-6 PUFA serum concentration (-0.809)<br>Omega-3 PUFA serum concentration (0.725)<br>Antioxidant score (0.589)	19.9
**Sum**		**71.2**

## Discussion

In recent decades, infertility has become increasingly prevalent in the human population, highlighting the need for research into potential biomarkers that could enhance our understanding and improve fertilisation success. Despite this growing need, there is still insufficient data to address the role of biomarkers in various pregnancy outcomes fully. Therefore, this study aimed to examine multiple factors potentially associated with pregnancy achievement.

Our study did not show significant differences in OS biomarkers between P and N groups of male and female patients undergoing ART. In line with these results, prooxidant, antioxidant, and oxy scores were also similar in the P and N groups. A review of the available literature revealed limited information on these biomarkers in couples undergoing the ART process [34] [35].

However, some studies on this topic indicate a predominantly negative role of OS markers in pregnancy achievement during the ART process [36]. A study investigating the relationship between serum TOS and TAS levels and clinical pregnancy outcomes in ART cycles found that TAS levels were significantly higher in patients who achieved clinical pregnancy in all four phases. Women with a higher TAS before and after oocyte retrieval and embryo transfer in ART cycles had an increased probability of achieving clinical pregnancy [37]. Additionally, Verit et al. [38] found that reduced PON1 activity may play a role in the pathogenesis of male subfertility. In contrast, our study did not find differences in PON1 between the P and N groups in males or females or other OS parameters. Nevertheless, a study by Beyazit et al. [39] reported that serum IMA levels in infertile women did not differ from those in healthy controls, which aligns with the results of our study.

The serum Zn concentration in our study was significantly higher in the men from the P group (787 μg/L) than in the N group (672 μg/L), despite no significant differences in the number of men taking Zn supplements, doses (10mg Zn/day), or the duration of supplementation (4–6 months). Reference values for Zn in serum are 11.2–25.9 μmol/L for males, that is 725–1275 μg/L, indicating Zn deficiency in the N group [40]. Studies suggest that Zn deficiency can negatively affect fertilisation success [41]. Zinc ions are associated with crucial processes in spermatozoa that are essential for acquiring fertilisation ability, including motility, capacitation, and acrosomal exocytosis. A low concentration of Zn permits normal sperm capacitation, enabling the development of hyperactivated motility, which culminates in a physiological acrosome reaction and successful fertilisation [6].

Although most patients in this study used vitamin (D) and mineral (Zn and Mg) supplements and antioxidants such as vitamin C or alpha-lipoic acid before the beginning of the ART procedure, there were no significant differences in supplements use between partners from the P or N group. The only observed difference was in antioxidant supplement use, which was higher among men in the P group than in the N group. Nevertheless, OS biomarkers in the blood were similar in both groups. Studies suggest that individuals can increase their fertilisation and ART success rates by enriching their diet with antioxidant substances [42].

A factorial analysis conducted on male subjects identified three distinct factors. The first factor predominantly encompassed seminogram parameters, which was anticipated given their common physiological origin.

The second factor identified in the analysis integrates vitamin (D) and mineral (Mg, Zn) intake with serum concentrations of PUFAs. A study by Niramitmahapanya et al. [43] specifically explored the relationship between vitamin D intake and FA levels in the blood. They found a positive association between monounsaturated fatty acids (MUFAs) and plasma levels of 25-hydroxyvitamin D. Conversely, they observed a negative association between vitamin D levels and PUFA concentrations, suggesting that higher vitamin D intake may influence FA composition in the blood by promoting MUFAs while reducing PUFA levels [43]. The relationship between Mg andvitamin D is important, as the conversion of vitamin D into its active form (calcitriol) requires Mg [44]. Additionally, both Mg and vitamin D play crucial roles in calcium metabolism.

Furthermore, Zn is involved in the PUFA metabolism, and dietary patterns related to Zn and PUFAsintake are associated with serum the linoleic (LA) / dihomo-linoleic acid (DGLA) ratio in males andfemales, which has been proposed as a novel biomarker of Zn status [45]. Zn and essential FA exhibit a synergistic relationship in many diseases; their deficiencies present similar clinical features [45]. Endogenous FA metabolism is regulated by delta-5 (D5), delta-6 (D6), and delta-9 (D9) desaturase enzymes, with Zn acting as a cofactor for desaturases and elongases in endogenous FA synthesis. Alterations in Zn levels may influence the activity of these enzymes, thereby impacting FA metabolism regulation [46]. Zn contributes to membrane flexibility, and a study by Hernandez et al. [47] reported a relationship between Zn supplementation and increased PUFAs in red blood cell membranes. In our study, we also identified a third factor: omega-6 PUFA levels in serum correlated with Zn status in both serum and seminal plasma, consistent with the relation between omega-3 PUFA with Zn, given the shared metabolic pathways for omega-3 and omega-6 PUFA.

The third factor in the male group connects serum concentrations of omega-6 PUFA and Zn withseminal Zn concentration and sperm pH value. Higher levels of Zn and other alkaline minerals areassociated with lower pH values, and a slightly alkaline testicular pH is conducive to spermatogenesis [48]. The molecular mechanisms underlying acidic pH conditions may directly damage the sperm cell membrane, potentially contributing to male infertility [49].

In the female cohort, the first factor, which involved the relationship between comprehensive redox status markers and the second factor regarding on vitamins/minerals intake, was not surprising. Excessive ROS production triggers OS, which is closely linked to female infertility. Our results confirmed this by revealing that the first factor, redox status, integrated the oxidative and prooxidant scores in relation to pregnancy achievement. A recent study found that an increase in oxidative balance score correlated with a decrease in female infertility, suggesting that heightened antioxidant levels and reduced prooxidant exposure might lower the risk of infertility in women [50]. The second factor in our study supports the role of minerals and vitamin intake in reducing infertility risk in women. Supplementing with micronutrients at least three months before ART cycles helps protect the follicular microenvironment from OS [51] [52]. Increased vitamin D intake positively correlates with fertility, while Mg facilitates the binding of folliclestimulating hormone to ovarian receptors [50]. In addition, Zn regulates various physiological processes of female germ cell growth, fertility and pregnancy.

The third factor included omega-3 and omega-6 PUFA concentration and antioxidant score level. As expected, omega-3 and omega-6 PUFA showed opposite dependence with antioxidant score, aligning with omega-3 concentration patterns. Although both omega-3 and -6 PUFA are prone to peroxidation, omega-3 PUFA are highly unsaturated and more susceptible to oxidation [31]. Serum PUFA levels depend on both metabolism and dietary intake. Although the role of PUFA intake and/or status in blood in ART outcome is not fully understood, a recent systematic review showed that omega-3 FAs might be beneficial by increasing the success rate of ART outcomes and improving embryo quality based on morphology and morphokinetic parameters [53].

The limitation of this study is the small sample size, particularly the number of men with different seminogram types. Another limitation is the use of supplements, which are not uniform and well controlled; however, these various supplementations are very common among men and women undergoing ART and are often not prescribed by medical doctors.

In summary, seminogram parameters, intake of micronutrients, and levels of Zn and omega-3 and omega-6 PUFA are contributing factors to ART success. To confirm their predictive role, the use of these biomarkers should be reassessed in a larger cohort. Moreover, high-quality randomised controlled trials are needed to evaluate the effects of different supplementations (omega-3 PUFA, vitamin D, Zn, antioxidants) on ART outcomes.

## Dodatak

### List of abbreviations

ART, assisted reproductive technology;<br>FA, fatty acids;<br>PUFA, polyunsaturated fatty acids;<br>PCA, principal component analysis;<br>Zn, zinc;<br>Mg, magnesium;<br>OS, oxidative stress;<br>BMI, body mass index;<br>AOPP, advanced oxidation protein products;<br>TOS, total oxidative status;<br>TMB, tetramethylbenzidine;<br>PAB, prooxidant-antioxidant balance;<br>IMA, ischemia-modified albumin;<br>SH, sulfhydryl;<br>DTT, dithiothreitol;<br>ABSU, absorption units;<br>TAS, antioxidative status;<br>ABTS, azino-bis sulfonic acid;<br>SOD, superoxide dismutase;<br>PON1, serum paraoxonase1;<br>SD, standard deviation;<br>SCD, desaturase activities;<br>ICP-MS, inductively coupled plasma mass spectrometry;<br>IQR, interquartile range.

### Author contributions

AV: original paper drafting, acquiring the data, formal analysis; JKS: interpreting the results, statistical analysis; AS: original paper drafting, interpreting the results; DBJ: sample collection; AA: formal analysis; MP: formal analysis, revising the manuscript; VV: supervision of the study, designing the work, interpreting the results, revising the manuscript. All authors approved the final version of the manuscript and agreed to be accountable for all aspects of the paper.

### Acknowledgements

This research was funded by the Ministry of Science, Technological Development and Innovation, Republic of Serbia (No. 451-03-47/2023-01/200015) and through two Grant Agreements with University of Belgrade-Faculty of Pharmacy No 451-03-65/2024-03/ 200161 and No 451-03-66/2024-03/ 200161.

### Conflict of interest statement

All the authors declare that they have no conflict of interest in this work.
